# Graft calcifications and dysfunction following liver transplantation

**DOI:** 10.1186/1471-2482-4-9

**Published:** 2004-09-03

**Authors:** George N Tzimas, Mohammad Afshar, Eric Chevet, Anouk Emadali, Hojatollah Vali, Peter P Metrakos

**Affiliations:** 1 Division of General Surgery/ Section of Hepatobiliary and Transplantation Surgery, Royal Victoria Hospital, McGill University Health Center, 687 Pine Avenue West, S 10.26, Montreal, Quebec, H3A 1A1, Canada; 2 Facility for Electron Microscopy Research, Strathcona Medical Building, 3640 University Street, McGill University, Montreal, Quebec, H3A 2B2, Canada; 3 Organelle Signaling Laboratory, Department of Surgery, Royal Victoria Hospital, H 6.34, McGill University, 687 Pine Avenue West, Montreal, Quebec, H3A 1A1, Canada

**Keywords:** Liver Transplantation, Ischemia, Reperfusion, Graft Dysfunction, Outcome

## Abstract

**Background:**

The molecular events, following ischemia and reperfusion (I/R) of the liver during transplantation are largely unknown. There is evidence that apoptotic and necrotic events may take place, and occasionally result in primary graft dysfunction. We herein report two cases, where significant I/R injury correlated with the development of liver calcification and primary liver dysfunction.

**Case Presentation:**

Both patients with clinical and biochemical evidence of primary graft dysfunction demonstrated calcification at light and electron microscopy levels. In addition, one patient had macroscopic evidence of calcification on cross-sectional imaging. Both patients died secondary to the sequelae of the graft dysfunction.

**Conclusions:**

Severe I/R-induced injury to the liver, clinically leads to graft dysfunction. This is due to advanced apoptotic and/or necrotic events at the hepatocyte level that may, on the most severe form, lead to calcification. The study of microcalcification at the early posttransplant period could provide insight in the events taking place following significant ischemia/reperfusion-induced injury to the graft.

## Background

Liver transplantation (LT) remains the only treatment for end-stage liver disease. However the transplant procedure mandates cold perfusion, hypothermic storage, warm ischemia and warm reperfusion of the graft, resulting in Ischemia/Reperfusion (I/R)-induced injury to the transplanted graft. Although the introduction of the University of Wisconsin solution (UW) has improved clinical outcomes, I/R injury remains one of the major clinical problems following LT leading, in cases of marginal donor quality or prolonged cold or warm ischemic time, to the development of graft dysfunction or non function.

Recently the cellular events following liver I/R during LT have been brought to sharp focus. Nevertheless, it is still controversial if the major mode of cell death during I/R is apoptosis and/or necrosis [1]. It has been shown in an animal model of viral-induced hemorrhagic liver necrosis, that the liver undergoes several morphologic changes including mineralization [2]. Furthermore, previous clinical reports have documented the development of calcifications in liver upon extensive ischemia [3,4]. The above observations may reflect a high degree of cell damage leading to an enhanced apoptotic cell engulfment by non-professional phagocytes of the liver [5] with subsequent necrosis and biomineralization.

In this report, using a combination of cross-sectional imaging, histochemistry, and light (LM) as well as transmission electron microscopy (TEM) we identified liver calcification, in two grafts with severe I/R injury following LT.

## Case Presentations

### Case 1

A 65-year-old man of Asian origin underwent LT at our institution on June 2002, for decompensated Hepatitis B related cirrhosis. The patient did not have a history of abnormal calcium metabolism, or hyperparathyroidism. Upon listing his serum ionized calcium was 1.07 mmol/L. The donor was a previously healthy, 42-year-old male, who suffered intracranial bleeding following a motor vehicle crash. He did not suffer any period of hypoxia but he was hypotensive (SBP = 90 mm Hg) prior to procurement. His serum liver function tests were normal prior to harvesting (AST 32 U/L, ALT 40 U/L). The organ was procured by our institution's transplant team. During procurement the liver was found to be well perfused with no focal injuries and no macroscopic evidence of steatosis.

The recipient underwent an uncomplicated conventional LT without the use of a veno-venous bypass. There were no periods of hypoxia or severe hypotension during transplantation. The cold ischemic time of the graft was 8 hours while the warm ischemic time was 45 minutes. The graft reperfused well and no biopsies were taken. Intravenous methylprednisolone (500 mg) was administered intraoperatively, and postoperatively the patient received induction with Antithymocyte Globulin, which is the protocol followed at our institution. During transplantation the patient received a total of 4 units of packed red blood cells (PRBCs) and 6 units of fresh frozen plasma (FFP).

**Figure 1:**
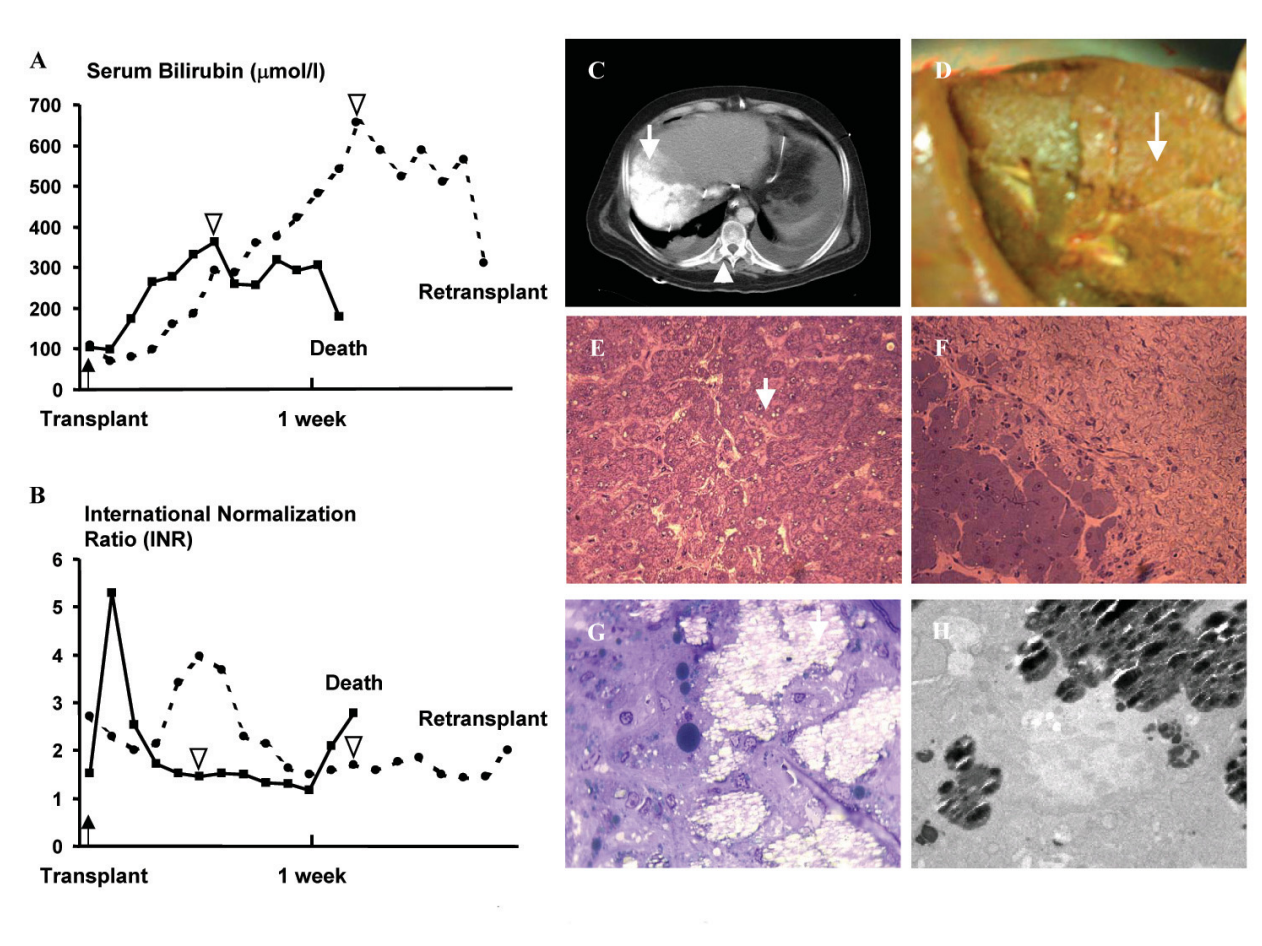
**A: **Serum Bilirubin levels (μmol/L) for patient 1 (solid line) and for patient 2 (dashed line). The arrowheads indicate the time points where calcifications were detected. **B: **International Normalization Ratio (INR) for patient 1 (solid line) and for patient 2 (dashed line). Arrowheads indicate the time points where calcifications were detected. **C: **Computed Tomography of patient 2. The arrow points an area in the right liver with the same density as the spinal column (arrowhead). **D: **Picture of the explant liver during retransplantation of the same patient (case 2). The arrow shows the abnormal area of the right liver correlating with the computed tomography findings. **E: **Light microscopy of epoxy embedded semi-section obtained from the liver biopsy from patient 1. The image shows moderate calcification (microcalcification) throughout the section. The arrow indicates a representative pattern of calcification. **F: **Light microscopy of epoxy embedded semi-section obtained from the tissues of the explant, following liver retransplantation of patient 2. The image shows the interface between calcified region (upper right) and non-calcified adjacent hepatic cells (lower left region). **G: **Light microscopy image showing a higher magnification of calcified region as shown in F. The bright and high contrast regions represent massive mineralization of hepatic cells of the explant, following retransplantation. **H: **Transmission electron microscopy images of ultrathin section obtained from the transitional zone between calcified and non-calcified tissue. Showing the mode of calcification and textural organization of hydroxyapatite crystal aggregates (dark contrast) within cytoplasmic region of the cell. Note alteration of the nucleus in the center.

At the time of LT, the international normalization ratio (INR) was 1.53, while serum total bilirubin was 103 μmol/L (Figure 1A,1B). On postoperative day 2 the patient had a peak of his serum AST (3469 U/L) and at this point he had further biochemical evidence of primary graft dysfunction, with inability to normalize his INR (Figure 1B), and with progressive elevation of his total serum bilirubin (Figure 1A). Repeated ultrasonographic examination revealed a patent hepatic artery and portal vein, as well as patent hepatic veins. At this point, a liver biopsy demonstrated severe reperfusion injury with several apoptotic bodies, several dystrophic calcifications (Figure 1E), and no evidence of acute cellular rejection. His clinical status deteriorated, he developed multiorgan system failure and died 12 days after his transplantation. No septic focus was identified. Both kidneys harvested from the same donor did not present any signs of delayed graft function after transplantation.

### Case 2

A 55-year-old man of Greek origin underwent LT at our institution for ethanol and hepatitis B related cirrhosis, as well as a large hepatocellular carcinoma. The patient did not have a history suggestive of hyperparathyroidism or abnormal calcium metabolism, and upon listing his serum ionized calcium was 0.94 mmol/L. The donor was a previously healthy, 52 year-old male who suffered a closed head injury during a motor vehicle crash. The organ was procured by our transplant team. Prior to procurement the donor did not suffer any periods of hypoxia or hypotension. His liver function tests were normal prior to harvesting (AST 36 U/L, ALT 45 U/L). During procurement the liver was found to be well perfused, with no evidence of aberrant vascular anatomy and no evidence of trauma. Macroscopic examination of the liver did not show any evidence of steatosis. The recipient underwent a conventional LT without venovenous bypass and without intraoperative hypotension. The cold ischemic time was 10 hours while the warm ischemic time was 40 minutes. Following reperfusion, the graft appeared well perfused and again no biopsies were taken. Intraoperatively the patient received 500 mg of methylprednisolone, and postoperatively he was induced with Antithymocyte Globulin. During transplantation the recipient received 3 units of PRBCs and 6 units of FFP. In the early postoperative period the patient had to be explored once for retroperitoneal bleeding associated with hypotension (SBP< 90 mm Hg).

At the time of LT, the patient had an INR of 2.7 and a serum total bilirubin of 104 μmol/L. Following LT, the patient developed severe primary graft dysfunction with rising serum bilirubin and INR (Figure 1A,1B), while his serum ALT and AST peaked during the 2^nd ^and 3^rd ^postoperative day (4700 U/L and 6598 U/L respectively). At that time, ultrasonography demonstrated uniformly patent vessels (hepatic artery, portal and hepatic veins), while Computed Tomography (CT) showed areas in the right lobe of the liver isodense with the spinal column (Figure 1C). The patient was listed for retransplantation. During the retransplant procedure the explant liver graft had a "bony" consistency in the involved right lobe. Cross-sections of the right lobe showed a "clay-like" parenchyma with clear evidence of calcification (Figure 1D). Light (LM) and transmission electron microscopy (TEM) investigation of ultrathin sections obtained from specimens selected from biopsies at the interface of calcified and non-calcified tissues showed extensive intracellular calcification within the hepatic cells (Figure 1F). High-resolution TEM (HRTEM) images and selected-area electron diffraction (SAED) combined with energy dispersive spectroscopy (EDS) analysis demonstrated the presence of hydroxyapatite (HA) as a solid phase in the calcified region. The adjacent non- or partially calcified hepatic cells displayed extensive nuclear condensation suggestive of significant apoptosis as well as severe vacuolization, suggestive of an extensive apoptotic and necrotic process (Figure 1G,1H).

The patient had a complicated postoperative course and finally died from ventricular fibrillation unresponsive to electrical cardioversion. Both kidneys harvested from the initial donor were transplanted without any evidence of delayed or primary graft dysfunction/non-function.

## Conclusions

Currently, more than 16,000 candidates are listed with the United Network for Organ Sharing awaiting liver transplantation. Nevertheless, only 4800 cadaveric liver transplants are performed annually in the United States. Due to this discordance between organ demand and supply, it is estimated that approximately 10% of patients in the waiting list will die before obtaining an organ. As a result, novel strategies to expand the donor pool have been explored. With the exception of live donor liver transplantation, the remaining strategies involve the use of older cadaveric grafts, allografts with mild steatosis or even donors with evidence of past hepatitis B or C infection [6]. This is why, one of the major obstacles to be tackled, is the development of clinically significant ischemia and reperfusion injury, which is even more important for "marginal" grafts. Every progress towards understanding the molecular events following not only cold storage but also cold and warm reperfusion of the graft could have a significant impact on the current transplantation practices. Indeed, recent data suggest that following I/R there is a balanced apoptosis and occasionally necrosis of hepatocytes translating into cell swelling, distension of various cellular organelles, clumping and random degradation of nuclear DNA, extensive plasma membrane endocytosis and autophagy [7]. Furthermore, when these events become predominant, they can lead, at least in animal models, to the development of calcifications as observed in livers of rabbits infected with rabbit haemorrhagic virus [2].

In the present case report, we have shown that these events can take place in human subjects following liver transplantation. To our knowledge these are the first reported cases in the literature of liver calcification following liver transplantation, presumably secondary to I/R injury. Not only did both patients have biochemical evidence of severe graft I/R injury, they also had biopsy proven I/R induced injury associated with the development of calcifications. Furthermore, both succumbed to the sequelae of this injury. Both recipients received grafts from donors with normal serum biochemistries and no evidence of hepatic trauma or steatosis. Both donors had no evidence of crystal deposition or storage disease, and although we did not perform any donor liver biopsies, the grafts appeared macroscopically normal and perfused well with UW solution. Corroborating to this remains the fact that all four kidneys (from both donors) were transplanted without any problems. Neither recipient had any evidence of calcium metabolism problems, since both had normal serum calcium upon listing. Both recipients had an anticipated intraoperative course without periods of hypotension and without massive transfusion requirements. Finally, both grafts did not demonstrate any vascular problems in the postoperative period by Ultrasonography or CT scan examination or any evidence of intrahepatic thrombosis in postmortem or explant examination.

Ischemic stress has been previously reported to induce calcium accumulation at the cell level, either by impaired energy metabolism and/or plasmalemmal alterations. This elevated intracellular calcium concentration is responsible for cytoskeletal modifications, which alter cell shape, and for the activation of phospholipases, which results in perpetuation of membrane damage and finally, mitochondrial calcification [8].

Although, the crystal shape, composition and organization of HA in our samples are similar to those observed in bone and cartilage [9], as well as synthetic HA formed in serum [10], the intracellular precipitation of HA within hepatic cells is unique and has not been reported from other physiological and pathological tissues.

The observation of calcified, vacuole-like structures in hepatic cells from these two livers could be suggestive of mitochondrial calcification. In addition, the extensive presence of phagocytic structures in the pre-calcified regions of these livers suggests an intense apoptotic/necrotic process undergone after I/R injury in these regions. Further investigation, however, is required to understand the mechanism(s) and the mode of calcification in the liver.

In conclusion, we believe that the described phenomenon is underreported at least in the liver transplant literature. Furthermore, it appears that there is a correlation between the development of severe I/R injury leading to apoptosis and/or necrosis and calcifications detectable even by light microscopy. We think that the development of microcalcifications should be studied more extensively in the context of I/R injury following liver transplantation. Although, such a phenomenon appears to correlate with significant I/R injury, evident by biochemical data, it has the potential to be provide further information on the pathways of severe I/R injury post transplant.

## Competing Interests

None declared.

## Authors Contributions

GNT: conceived the study and wrote the manuscript

MA: carried out the electron microscopy studies

EC: participated in the design and analyzed the light microscopy results, critical review of the manuscript

AE: analyzed light microscopy results

AV: carried out electron microscopy studies

PM: participated in the design of the study

All authors read and approved the final manuscript.

## Abbreviations

**I/R: **Ischemia and Reperfusion

**LM: **Light Microscopy

**TEM: **Transmission Electron Microscopy

**HRTEM: **High-resolution Transmission Electron Microscopy

**EDS: **Energy Dispersive Spectroscopy

**HA: **Hydroxyapatite

## Pre-publication history

The pre-publication history for this paper can be accessed here:


